# Proteomic analysis shows that stress response proteins are significantly up-regulated in resistant diploid wheat (*Triticum monococcum*) in response to attack by the grain aphid (*Sitobion avenae*)

**DOI:** 10.1007/s11032-015-0220-x

**Published:** 2015-01-28

**Authors:** Wenzhu Guan, Natalie Ferry, Martin G. Edwards, Howard A. Bell, Hamizah Othman, John A. Gatehouse, Angharad M. R. Gatehouse

**Affiliations:** 1 Newcastle Institute for Research on Sustainability, School of Biology, Newcastle University, Newcastle upon Tyne, NE1 7RU UK; 2 School of Environment and Life Science, Salford University, Salford, M5 4WT UK; 3 The Food and Environment Research Agency, Sand Hutton, York, YO41 1LZ UK; 4 School of Biological and Biomedical Sciences, University of Durham, Durham, DH1 3LE UK

**Keywords:** Aphid resistance, Biotic stress, Diploid wheat, Oxidative stress, Stress response, Wheat proteomics

## Abstract

The grain aphid *Sitobion avenae* (F.) is a major pest of wheat, acting as a virus vector as well as causing direct plant damage. Commonly grown wheat varieties in the UK have only limited resistance to this pest. The present study was carried out to investigate the potential of a diploid wheat line (ACC20 PGR1755), reported as exhibiting resistance to *S. avenae,* to serve as a source of resistance genes. The diploid wheat line was confirmed as partially resistant, substantially reducing the fecundity, longevity and growth rate of the aphid. Proteomic analysis showed that approximately 200 protein spots were reproducibly detected in leaf extracts from both the resistant line and a comparable susceptible line (ACC5 PGR1735) using two-dimensional gel electrophoresis and image comparison software. Twenty-four spots were significantly up-regulated (>2-fold) in the resistant line after 24 h of aphid feeding (13 and 11 involved in local and systemic responses, respectively). Approximately 50 % of all differentially expressed protein spots were identified by a combination of database searching with MS and MS/MS data, revealing that the majority of proteins up-regulated by aphid infestation were involved in metabolic processes (including photosynthesis) and transcriptional regulation. However, in the resistant line only, several stress response proteins (including NBS–LRR-like proteins) and oxidative stress response proteins were identified as up-regulated in response to aphid feeding, as well as proteins involved in DNA synthesis/replication/repair. This study indicates that the resistant diploid line ACC20 PGR1755 may provide a valuable resource in breeding wheat for resistance to aphids.

**Electronic supplementary material:**

The online version of this article (doi:10.1007/s11032-015-0220-x) contains supplementary material, which is available to authorized users.

## Introduction

Wheat, *Triticum aestivum* L., is currently second to rice as the main human food crop and is the leading source of vegetable protein in human nutrition (United Nations 2012). Aphids (Order Hemiptera) are major insect pests of world agriculture, damaging crops by removing photoassimilates and vectoring numerous plant viruses (Smith and Boyko 2007). The grain aphid, *Sitobion avenae* (Fabricius), is considered a serious pest of commercial wheat in the UK. Many aphid species have evolved resistance to insecticides (Devonshire and Field 1991), and restrictions on the availability of active ingredients for insecticide production in Europe (European Directives 91/414/EEC) have prioritised research on crop varieties with resistance to aphid pests (Smith 2005). Most commercial wheat varieties have very little resistance to aphid pests (Migui 2002; Migui and Lamb 2003), with at best partial antibiosis, antixenosis and tolerance in some winter varieties (Havlícková 1993). However, recently a synthetic wheat line (98-10-35) with moderate levels of resistance to the grain aphid has been developed, although the mechanism of this constitutive resistance to *S. avenae* is not known (Wang et al. 2013).

Aphids differ from many other pest insects in their mode of feeding in that they do not cause major tissue damage or induce plant wounding responses (Farmer and Ryan 1992; Gatehouse 2002) since they feed from plant phloem by inserting a stylet between the cells. As a result, plant responses to aphid feeding have been reported to be similar to those induced by pathogen attack and, in some cases, have been classified as gene-for-gene interactions characteristic of plant–pathogen interactions (Walling 2000; Moran and Thompson 2001; Moran et al. 2002; Smith and Boyko 2007). This response has been reported to be induced by aphid-derived elicitors with salicylic acid (SA) as the major signalling molecule (Walling 2000; Moran et al. 2002; Smith and Boyko 2007). However, aphids can also induce a non-specific defence response in the plant, resulting in a general stress response, which can be detected in both aphid-resistant and aphid-susceptible plants (Smith and Boyko 2007). Plants experience extensive transcriptome reprogramming when they are subjected to insect attack (Moran and Thompson 2001; Zhang et al. 2004; De Vos et al. 2005; Yuan et al. 2005; Wei et al. 2009). Ferry et al. (2011) demonstrated that the proteome of wheat, variety ‘Claire’ a commercial winter wheat cultivar commonly grown in the UK, changed following aphid infestation. These changes were more consistent with SA-induced responses than the jasmonic acid (JA)-induced wounding responses, although none of these were sufficient to confer resistance to the grain aphid. These findings confirm previous studies where rice brown planthopper (*Nilaparvata lugens*) caused changes in the expression of rice proteins involved in signalling pathways, oxidative stress/apoptosis, wound response, drought response and pathogen-related response (Zhang et al. 2004).

Whilst none of the commercial wheat varieties grown in Europe are resistant to *S. avenae* or other cereal aphids of European origin (Di Pietro et al. 1998; Migui 2002; Migui and Lamb 2003), some commercial wheat has partial resistance towards Russian wheat aphids, *Diuraphis noxia* (Mordvilko) (RWA). Ten RWA resistance genes have been identified in *T. aestivum* and named *Dn* genes (Ma et al. 1998). Microarray and real-time PCR analysis revealed that more than 180 genes up-regulated on attack by *D. noxia* are related to reactive oxygen species, signalling, pathogen defence and arthropod allelochemical and physical defence (Smith et al. 2010). In a further study, superoxide dismutase, glutathione reductase and ascorbate peroxidase were uniquely up-regulated in the RWA resistant wheat (*T. aestivum*) cultivar Tugela DN, compared to the RWA susceptible cultivar Tugela after *D. noxia* infestation. These findings suggest the involvement of antioxidative enzymes in the RWA–wheat resistance response to minimise toxic effects to plant cells (Moloi and van der Westhuizen 2006, 2008).

Modern wheat varieties are hexaploid and have low genetic diversity for insect resistance traits (Ogbonnaya et al. 2013; Donini et al. 2000). A screen of 87 ancient diploid wheat genotypes, including 67 *Triticum monococcum* L. genotypes, 13 *Triticum boeoticum* Bois genotypes, 7 *Triticum urartu* Tumanian ex Gandilyan genotypes, showed that many exhibited higher resistance to *S. avenae* than a cultivar of the modern wheat *T. aestivum* (variety ‘Arminda’; Di Pietro et al. 1998). Accessions of the diploid ancestral wheat—Einkorn wheat, *T. monococcum* with partial resistance to aphids—have been reported (Migui and Lamb 2004). Furthermore, accessions of wild wheat species, *T. boeoticum* (Bois)*, T. tauschii* (Coss.) *Schmal.* and *T. araraticum* Jakubz., were also found to exhibit high levels of resistance to aphids (Migui and Lamb 2003). Thus, ancient diploid wheat may be a useful source of genes for improving resistance to *S. avenae* in modern wheat (Di Pietro et al. 1998). However, no previous studies have been carried out to investigate differential gene expression in these lines in response to aphid infestation. The aim of this study was therefore to identify putative defence genes in diploid wheat lines (*T. monococcum*) in response to grain aphid (*S. avenae*) feeding using differential proteomics to better understand the basis of this observed resistance/tolerance.

## Materials and methods

### Plant materials and treatment

Two diploid accessions of wild Einkorn wheat, ACC5 PGR1735 and ACC20 PGR1755, were selected for study, representing lines exhibiting aphid susceptibility and tolerance, respectively. These seeds were kindly donated by R. J. Lamb from seed collections of Agriculture and Agri-Food Canada. They are described as ‘accessions’ in Migui and Lamb (2004). Wheat seedlings were grown to the four-leaf stage in soil (John Innes, No. 2) under controlled environmental conditions in custom-built growth rooms (HA Davie Ltd, U.K.) under the following conditions (light intensity; photosynthetically active photon flux: 600 mol/m^2^/s, 16:8 LL:DD light regime) with a temperature regime of 18 °C (day):16 °C (night) and 70 % relative humidity.

### Aphid bioassays

Grain aphids, *S. avenae*, were obtained from a laboratory culture and maintained at 20 °C, 55 % relative humidity (R.H.) under a 16:8 LL:DD light regime. The aphids were reared on oats (*Avena sativa* L., cv. Coast Black), and infested plants were kept in 45 × 45 × 50 cm Perspex cages with new plant material supplied weekly.

**Fig. 1:**
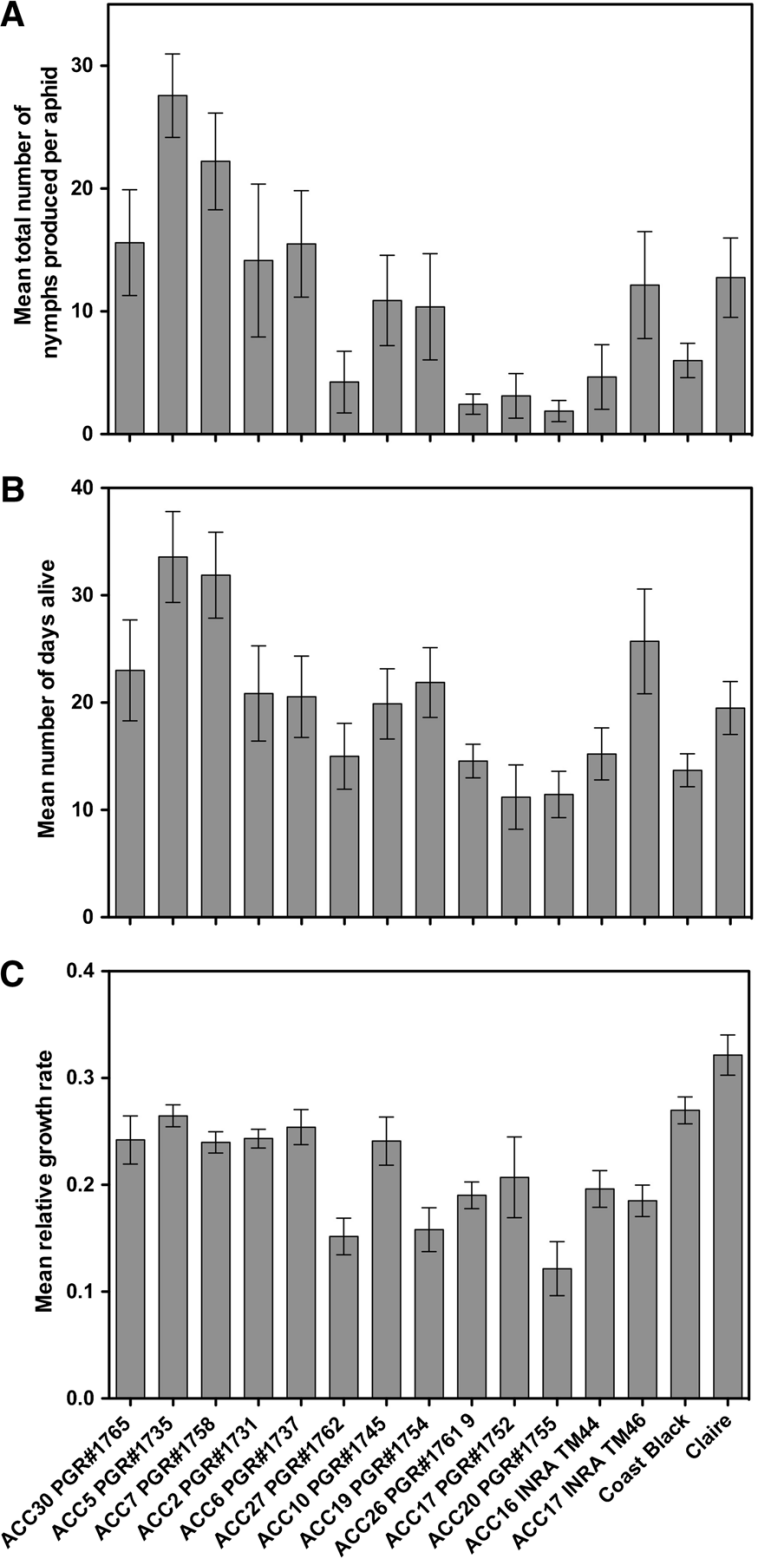
Thirteen accessions of diploid Einkorn wheat lines together with an oat line (cv. Coast Black) and a hexaploid wheat reference line (cv. Claire) were screened for aphid (*Sitobion avenae*) resistance using three parameters: mean fecundity (**a**), longevity (**b**) and relative growth rates (**c**). Based on this bioassay data, a resistant (R, ACC20 PGR1755) and a susceptible line (S, ACC5 PGR1735) were selected for further study to investigate potential genes involved in aphid tolerance/resistance using a reverse genetic approach

Growth, survival and fecundity of *S. avenae* were evaluated on thirteen accessions of *Triticum monoccoccum* diploid wheat (Fig. 1). Experiments were also set up using a hexaploid wheat cultivar (Claire) and oats (Coast Black as comparisons). Single seedlings, at the two-leaf stage, of each wheat cultivar and oats were planted in 9-cm pots containing John Innes No. 3 compost and allowed to establish for 2–3 days. To assess fecundity and longevity, two-leaf stage seedlings of the different wheat varieties were each infested with one neonate nymph and randomly arranged within a growth room 20 °C, 55 % R.H. under a 16:8 LL:DD light regime. On each day following infestation, the survival of aphids was monitored, and following ecdysis to the adult stage, the production of nymphs was recorded. Ten replicates were set up for each plant type. To determine mean relative growth rates (MRGRs), neonate nymphs were removed from culture using a fine camel hair paintbrush and weighed on a Mettler AT20 balance. The aphids were then transferred singly to a seedling of one of the wheat varieties or oats. Following infestation, the plants were enclosed in 25 × 9 cm ventilated plastic cylinders to prevent aphid escape and arranged randomly within a growth room maintained at the conditions detailed above. The nymphs were reweighed after 4 days and again when they had moulted to the adult. The mean relative growth rates of aphids developing on the different plants, and over different time scales, were calculated using the methods described by Leather and Dixon (1984).

Data were analysed using one-way analysis of variance (ANOVA). Means were subsequently separated by Tukey–Kramer I tests. Analysis was conducted using GraphPad Prism 5.0.

### Aphid infestation for proteomic experiments

The set-up and protocol for aphid infestation on both resistant and susceptible wheat plants (local and systemic treatments) were the same as the ones used by Ferry et al. (2011). In brief, wheat seedlings (four-leaf stage) were infested with *S. avenae* adults (20 aphids per leaf) confined to two leaves with clip cages (12 plants per treatment). There were 8 treatments in total in this study, as illustrated in the table:

**Table:** 

	Time of infestation	Time of infestation
Wheat line	24 h	8 days
Resistant accession	Local	Local
	Systemic	Systemic
Susceptible accession	Local	Local
	Systemic	Systemic

Aphids were left on the seedlings for either 24 h or 8 days after which time they were removed and leaf tissues immediately frozen in liquid nitrogen. Tissues exposed to aphids were designated ‘local treatment’ and the leaves not infested on the same plant designated ‘systemic treatment’. Corresponding tissues were taken from aphid-free control plants for all time points and treatments.

### Protein extraction

Leaf samples from 12 experimental plants (4-leaf stage, divided into local and systemic infested tissues) and six control plants were ground to a fine powder using a mortar and a pestle with liquid nitrogen. Samples were pooled into three biological replicates per treatment; six technical replicates of the pooled samples were used in this work. Protein extraction, resuspension, quantification, IEF, SDS-PAGE and staining were carried out following the protocol used in Ferry et al. (2011). In brief, samples were incubated in 10 % (w/v) trichloroacetic acid/acetone with 0.07 % v/v 2-mercaptoethanol at -20 °C for 5 h and then centrifuged at 35,000×*g* for 20 min. The pellets were washed with ice-cold acetone (0.07 % 2-mercaptoethanol) incubated at −20 °C for 1 h and centrifuged at 12,000×*g* for 15 min. This wash was repeated six times. Pellets were vacuum dried, and the resultant powder suspended in 7 M urea, 2 M thiourea, 4 % CHAPS, 60 mM DTT, 0.5 % v/v pH 3–10 carrier ampholytes and 1 % v/v protease inhibitor mix (GE Healthcare, Bucks, UK) by sonication. Centrifugation at 20,000×*g* for 20 min at 4 °C removed insoluble debris, and the total protein content of the supernatant was determined using the 2-D Quant kit (GE Healthcare) method with BSA as standard. Isoelectric focussing (IEF) and SDS-PAGE were carried out essentially according to the manufacturers’ instructions on an Ettan IPGphor II and Ettan DALTsix system (GE Healthcare). For IEF, 18-cm IPG strips with a nonlinear gradient (pH 3–10) were actively rehydrated using 340 μl of DeStreak Rehydration solution (GE Healthcare) at 30 V for 10 h. Five hundred micrograms of protein from each pooled sample was loaded, and IEF conducted at 20 °C under the following conditions: 500 V for 500 Vh, followed by two gradients of 1,000 V for 800 Vh, 8,000 V for 13,500 Vh and finally 8,000 V for 20,000 Vh. The focussed strips were equilibrated for 15 min in 10 mL equilibration solution (75 mM Tris–HCl, 6 M urea, 30 % w/v glycerol, 2 % w/v SDS, 1 % w/v DTT) followed by equilibration in buffer containing 3 % w/v iodoacetic acid for another 15 min. Strips were transferred to a vertical SDS-PAGE gel, and the second dimension performed on a 12.5 % gel using the Ettan DALTsix system (GE Healthcare) at 4 °C. Protein spots were stained with colloidal Coomassie blue G-250 (Sigma).

### Image and data analysis

Wet stained gels were scanned using a LabScan 5.0 (GE Healthcare) at a resolution of 600 dpi, bit depth 12. Image analysis, spot detection and quantification were carried out using the Progenesis SameSpots software package version 4.0 and 4.1 (Nonlinear Dynamics, Newcastle, UK). Image and data analysis were as previously described (Ferry et al. 2011). Total spot volume (from matched gel replicates) for each treatment was compared to control images, and a threshold level of ±2-fold change set and subjected to statistical analysis using ANOVA. Spots were determined to be significantly up- or down-regulated when *p* value was <0.05 (Tukey–Kramer post hoc test). Within group comparisons allowed quality control of images. The molecular masses of protein spots were determined by co-electrophoresis of Mark 12 standard protein markers (Invitrogen), and pI values estimated from the Immobiline DryStrip as per manufacturers recommendations and further calibrated by comparison to published wheat leaf proteomes (Ferry et al. 2011).

### MALDI-TOF MS, MS/MS and database search

Selected protein spots from 2D gels with two changes in spot volume after aphid infestation were excised from gel, digested with sequencing grade trypsin (Promega, USA) and subjected to MALDI-TOF MS and MS/MS combined with database searching to assign putative identities to the proteins, as previously described (Ferry et al. 2011). Tryptic peptides were deposited onto 384-well stainless steel target plates manually with a calibration mix (Peptide calibration standard; Bruker Daltonics, Germany) spotted between every 4 sample wells and overlaid with 0.5 μl matrix (CHCA). Peptide mass spectra were recorded using a Bruker UltraFlex II MALDI mass spectrometer in positive reflectron mode over a m/z range of 700–4,500 and analysed using the instruments Flex Analysis software (v3.0). Monoisotopic peak selection was restricted to 50 peptides, and known trypsin autolysis peaks deleted, the peak list was searched against the nrNCBI and Swiss-Prot databases restricted to ‘Viridiplantae’ using the MASCOT (www.matrixscience.com) search engine or a local custom-built restricted database constructed from freely available EST libraries (8,530 wheat and 7,341 barley, http://trifldb.psc.riken.jp/index.pl) with the MASCOT 2.2 software allowing an m/z error of 50 ppm, maximum two missed cleavages, and possible modification of cysteines by carbamidomethylation as well as oxidation of methionine. Protein identification was accepted based on a significant MOWSE score and at least four matched peptide masses. Matching ESTs were queried to the nrNCBI database with a significance cut-off value of 1e-5 using BLASTX searches. Gene ontology (GO) phrases were collected using the PRO protein ontology search engine (http://pir.georgetown.edu/pro/) using the generic GO Slim. Putative protein ID and gene annotation are listed in Tables 1 and 2.

**Table 1:** Identification by PMF of differentially regulated proteins showing a minimum twofold increase in abundance in response to *S. avenae* infestation of resistant or susceptible diploid lines at 24-h time point in local and systemic tissues

Spot Name (a)	Ac. No. (b)	Predicted ID (c)	Score (d)	e-Value (e)	Th. pI (f)	Function and biological process/category (g)
*RL versus RC 1*	Q56YM6	Hypothetical protein—*Arabidopsis thaliana*	69	0.0075	8.88	2-Polyprenyl-6-methoxyphenol hydroxylase and related FAD, involves in oxidation–reduction process—response to oxidative stress
*RL versus RC 2*	Q6K6S5	Heat stress transcription factor A-5—*Oryza sativa*	52	0.015	5.3	Regulation of transcription/response to stress—stress response (and transcriptional regulation)
*RL versus RC 3*	Q84R82	Hypothetical protein OSJNBb0041J20.14.—*Oryza sativa*	53	0.39	11.19	Unknown—Unknown
*RL versus RC 4*	Q53P41	Lipase class 3 family protein, putative, expressed—*Oryza sativa*	63	0.031	8.62	Triacylglycerol lipase activity—other metabolism (lipase)
*RL versus RC 5*	Q7YMR6	Ribulose-1,5-bisphosphate carboxylase/oxygenase large subunit—*Hordeum vulgare*	93	0.00012	6.22	Ribulose bisphosphate carboxylase complex; ribulose bisphosphate carboxylase activity—photosynthesis
*RL versus RC 6*	B5B1F8	Chloroplast aspartate aminotransferase—*Triticum aestivum*	58	0.97	6.69	L-aspartate: 2-oxoglutarate aminotransferase activity; pyridoxal phosphate binding—photosynthesis
*RL versus RC 7*	Q09QZ5	ATPase alpha subunit—*Tetraphis pellucida*	57	1.2	9.21	Hydrogen ion transporting ATP synthase activity, rotational mechanism/proton-transporting ATPase activity—ATP synthesis
						Dephosphorylation reaction, releases energy—ATP synthesis
*RL versus RC 8*	Q8L5E7	Dof zinc finger protein (Fragment)—*Hordeum vulgare*	52	1.6	9.84	Transcriptional factor (activator/repressor), may permit leaf-specific gene expression—transcriptional regulation
*RL versus RC 9*	B23703	Ribulose bisphosphate oxygenase carboxylase activase—*Hordeum vulgare*	97	4.9E_05	5.64	ATP binding, nucleotide binding, chloroplast component, ligase activity, have nucleotide-binding motif A (P-loop)—photosynthesis
	C23703	Ribulose bisphosphate oxygenase carboxylase activase—*Hordeum vulgare*	95	7.7E_05	8.39	ATP binding, nucleotide binding, chloroplast component, ligase activity, have nucleotide-binding motif A (P-loop)—photosynthesis
	Q40073	Ribulose bisphosphate carboxylase/oxygenase activase A—*Hordeum vulgare*	84	0.9E_03	8.62	Chloroplast stroma component, ATP binding. Activation of RuBisC—photosynthesis
*RL versus RC 10*	Q10EH0	Lipoxygenase—*Oryza sativa*	59	0.33	6.82	Lipoxygenase activity—other metabolism (Lipoxygenase)
*RL versus RC 11*	B23703	Ribulose bisphosphate oxygenase carboxylase activase—*Hordeum vulgare*	104	9.9E_06	5.64	ATP binding, nucleotide binding, chloroplast component, ligase activity, have nucleotide-binding motif A (P-loop)—photosynthesis
	C23703	Ribulose bisphosphate oxygenase carboxylase activase—*Hordeum vulgare*	100	2.5E_05	8.39	ATP binding, nucleotide binding, chloroplast component, ligase activity, have nucleotide-binding motif A (P-loop)—photosynthesis
	Q40073	Ribulose bisphosphate carboxylase/oxygenase activase A-*Hordeum vulgare*	86	0.62E_03	8.62	Chloroplast stroma component, ATP binding. RuBisCo activase—photosynthesis
*RL versus RC 12*	Q76MG1	Dehydrin—*Nicotiana tabacum*	64	0.25	5.62	Response to stress, response to water—stress response
*RL versus RC 13*	A2WZ04	Putative uncharacterised protein—*Oryza sativa*	59	0.77	7.22	Unknown—unknown
RS versus RC 2	A9T3C3	Predicted protein—*Tetraphis pellucida*	63	0.35	7.05	Unknown—unknown
RS versus RC 3	Q69X41	Putative uncharacterised protein P0429G06.11—*Oryza sativa*	55	4.3	12.05	Unknown—unknown
RS versus RC 5	P12782	Phosphoglycerate kinase, chloroplastic—*Wheat*	59	0.32	6.58	Kinase and transferase, involves in carbohydrate biosynthesis/calvin cycle—photosynthesis
	O48991	NBS–LRR type resistance protein—*Oryza sativa*	53	2.7	5.68	ATP binding, involves in apoptosis and defence response-Apoptosis and defence response
RS versus RC 6	Q7EZQ0	OJ1005_b10.1 protein—*Oryza sativa*	54	5.2	11.17	Unknown—unknown
RS versus RC 7	A9T2E0	Predicted protein—*Tetraphis pellucida*	58	0.98	10.02	Unknown—unknown
RS versus RC 8	B9IMQ9	Predicted protein—*Black cottonwood*	57	1.3	8.81	Unknown—unknown
RS versus RC 9	Q7F7Z1	Hypothetical protein—*Oryza sativa*	59	0.79	11.56	Unknown—unknown
RS versus RC 10	B6TSE5	Ras-related protein Rab-18—*Zea Mays*	52	3.7	6.34	Small GTPase superfamily, Rab family. GTP binding and nucleotide binding—other metabolism (GTPase)
RS versus RC 12	XP_002266213	Hypothetical protein—*Vitis vinifera*	67	0.13	9.22	Unknown—unknown
RS versus RC 13	Q6YTS9	Os08g0484700 protein (proline-rich family protein-like)—*Oryza sativa*	54	0.57	7.09	Unknown—unknown
RS versus RC 15	Q84YU2	Putative uncharacterised protein P0045A07.118—*Oryza sativa*	57	2.4	11.2	Unknown—unknown
**SL versus SC 5**	Q6L5A9	Os05g0545500 protein—*Oryza sativa*	75	8.60E−03	10.59	Unknown—unknown
**SL versus SC 7**	Q655S1	Cell division protease ftsH homolog 2, chloroplastic—*Oryza sativa*	86	5.50E−04	5.54	Probable ATP-dependent zinc metallopeptidase, have hydrolase, metalloprotease and protease activities—cell division (peptidase/protease)
**SL versus SC 8**	Q8S6F3	ATP synthase subunit beta—*Oryza sativa*	95	8.40E−05	5.38	Hydrogen ion transporting ATP synthase activity, rotational mechanism—ATP synthesis
**SL versus SC 9**	P00828	ATP synthase subunit beta, chloroplastic—*Hordeum vulgare*	102	1.60E−05	5.11	Produces ATP from ADP in the presence of a proton gradient across the membrane—ATP synthesis
**SL versus SC 13**	Q6Z9W5	Epstein-Barr virus EBNA-1-like protein—*Oryza sativa*	68	0.04	11.42	Unknown—unknown
**SL versus SC 14**	Q3T5J7	Ribulose bisphosphate carboxylase large chain—*Sapium glandulosum*	72	0.014	6.05	Calvin cycle, carbon dioxide fixation and photosynthesis, have lyase, monooxygenase and oxidoreductase activities—photosynthesis
**SL versus SC 18**	A9TYL4	Predicted protein—*Physcomitrella patens*	82	4.70E−03	6.52	Unknown—unknown
**SL versus SC 22**	Q75K85	Putative uncharacterised protein—*Oryza sativa*	73	0.012	6.28	Chromosome organisation, chromosome, ATP-binding and protein binding activities—chromosome organisation
**SL versus SC 25**	Q9SEU4	At1g55310—*Arabidopsis thaliana*	82	1.60E−03	11.73	Nuclear mRNA splicing, via spliceosome, identical protein binding, nucleic acid-binding and nucleotide-binding activities—mRNA processing
**SL versus SC 27**	Q9C7P1	Putative zinc finger CCCH domain-containing protein 10—Arabidopsis thaliana	64	0.011	8.74	DNA binding and zinc ion binding—unknown
**SL versus SC 15**	P12782	Phosphoglycerate kinase, chloroplastic—Triticum aestivum	74	0.026	6.58	Involves in glycolysis; ATP-binding and phosphoglycerate kinase activities—other metabolism (glycolysis)
**SL versus SC 26**	Q75G97	Putative uncharacterised protein OSJNBa0042F15.18—Oryza sativa	81	0.006	7.61	DNA integration and RNA-dependent DNA replication, DNA-binding, RNA-binding and RNA-directed DNA polymerase activities—DNA processing
***SS versus SC 9***	P00828	ATP synthase subunit beta, chloroplastic—Arabidopsis thaliana	74	9.40E−03	5.11	ATP synthesis, hydrogen ion transport, hydrolase activity (catalyse hydrolysis)—ATP synthesis
***SS versus SC 13***	Q9FQ02	5′-3′ exoribonuclease 2—Arabidopsis thaliana	67	6.30E−03	9.04	mRNA processing and miRNA catabolic process, 5′-3′ exoribonuclease activity, nucleic acid binding and zinc ion binding—mRNA and miRNA processing
***SS versus SC 4***	Q69LV5	Putative uncharacterised protein OSJNBa0057D11.41—Oryza sativa	75	7.80E−03	11.72	Unknown—unknown
***SS versus SC 8***	Q67H50	ATP synthase beta subunit (Fragment)—Sisyrinchium montanum	72	0.017	5.28	Produces ATP from ADP in the presence of a proton gradient across the membrane—ATP synthesis

Keys: (above proteins are arranged by treatments)

Italic: proteins up-regulated in Local tissues of Resistant line to 24-h aphid infestation

Normal: proteins up-regulated in Systemic tissues of Resistant line to 24-h aphid infestation

Bold: proteins up-regulated in Local tissues of Susceptible line to 24-h aphid infestation

Bold italic: proteins up-regulated in Systemic tissues of Susceptible line to 24-h aphid infestation

Some proteins have more than one eligible ID, and it is not possible to distinguish which ID is true due to limitations of the approach

**Table 2:** Identification by PMF of differentially regulated proteins showing a minimum twofold increase in abundance in response to S. avenae infestation of resistant or susceptible diploid lines at 8-day time point in local and systemic tissues

RL versus RC 1Spot Name (a)	Ac. No. (b)	Predicted ID (c)	Score (d)	e-Value (e)	Th. pI (f)	Function and biological process/category (g)
*RL versus RC 1*	Q5NB00	Putative uncharacterised protein P0483F08.25O—Oryza sativa	57	4.8	11.92	Unknown—unknown
*RL versus RC 2*	Q94HR4	Hypothetical protein OSJNBa0065C16.5—Oryza sativa	66	2	11.95	Unknown—unknown
*RL versus RC 3*	Q7XYU5	Integrase (fragment)—Gossypium herbaceum	62	0.16	9.24	DNA binding—unknown
*RL versus RC 4*	Q6RH10	60S ribosomal protein L13a—Capsicum annuum	68	0.041	10.83	Translation, ribonucleoprotein, structural constituent of ribosome—protein synthesis
*RL versus RC 5*	Q1SZU6	RNA-directed DNA polymerase—Drosophila melanogaster	63	0.12	10.06	Unknown—unknown
*RL versus RC 6*	Q6XRD3	Ribosomal protein S4 (Fragment)—Passiflora morifolia	77	5.10E−03	10.28	Translation, ribonucleoprotein, structural constituent of ribosome, chloroplast—protein synthesis
*RL versus RC 7*	Q9MBH3	Arabidopsis thaliana genomic DNA—Arabidopsis thaliana	59	0.033	9.74	Unknown—unknown
*RL versus RC 8*	Q7XXI2	OSJNBa0059H15.7 protein P- Oryza sativa	135	7.8E−09	8.94	DNA integration, DNA binding—unknown
	Q7Y008	Putative RNA-binding protein—Oryza sativa	135	7.8E−09	9.05	nucleic acid binding, Zn ion binding—unknown
	Q1S1F3	Polyprotein—Medicago truncatula	68	0.041	9.84	Unknown—unknown
*RL versus RC 9*	Q9FNL5	Similarity to guanylate binding protein—Arabidopsis thaliana	86	6.40E−04	6.25	Immune response/beta-lactamase activity, GTP binding—immune response
*RL versus RC 11*	Q3HRW1	60S ribosomal protein L13a-like protein—Solanum tuberosum	66	0.064	10.54	Translation, ribonucleoprotein, structural constituent of ribosome—protein synthesis
*RL versus RC 12*	Q9SL02	DNA repair protein RAD50—Arabidopsis thanliana	57	0.53	5.98	DNA repair, Mre11 complex, ATP binding, nuclease activity—DNA processing
*RL versus RC 13*	Q8H636	Os06g0128100 protein—Oryza sativa	129	3.10E−08	7.85	Putative methyltransferases—other metabolism (Methyltransferase)
	Q10JM0	Retrotransposon protein, putative, Ty3-gypsy subclass—Oryza sativa	129	3.10E−08	8.92	RNA-dependent DNA replication—DNA processing
*RL versus RC 14*	Q6H660	Putative stress-induced protein sti1—Oryza sativa	58	0.41	6.03	Heat-shock chaperonin-binding—stress response
*RL versus RC 15*	Q8GWT6	Putative uncharacterised protein At1g53885/T18A20.20 (At1g53885)—Arabidopsis thaliana	74	0.009	9.57	Unknown—unknown
*RL versus RC 16*	Q2QLW7	Retrotransposon protein, putative—Oryza sativa	62	0.15	8.88	RNA-dependent DNA replication, RNA-directed DNA polymerase activity—DNA processing
*RL versus RC 17*	S43767	Ribosomal protein S3—evening primrose mitochondrion—Oenothera villaricae	69	0.03	10.24	Unknown—unknown
*RL versus RC 18*	P36688	50S ribosomal protein L12, chloroplastic—Nicotiana sylvestris	54	2.1	5.99	Translation, ribonucleoprotein, structural constituent of ribosome—protein synthesis
*RL versus RC 19*	Q84M91	Putative uncharacterised protein At3g05330—Arabidopsis thaliana	63	0.12	11.7	Cytokinesis, initiation of separation, microtubule associated complex—cytokinesis
*RL versus RC 20*	Q7XPG9	OSJNBb0003B01.14 protein—Oryza sativa	68	0.035	6.02	Cysteine-type peptidase activity—peptidase
*RL versus RC 21*	Q5Z660	Putative viral resistance protein—Oryza sativa	53	1.4	6.17	Apoptosis, ATP binding—apoptosis and defence response to virus
*RL versus RC 22*	Q8H3G8	Myosin heavy chain-like protein—Oryza sativa	66	0.7	5.55	Unknown—unknown
*RL versus RC 23*	Q9FVL0	Non-symbiotic haemoglobin 1, MEDsa GLB1—Medicago sativa	59	0.34	9.08	Stress response/NsHb/non-symbiotic haemoglobin, heme-binding, oxygen-binding—stress response
*RL versus RC 24*	Q9AUR0	Putative uncharacterised protein OSJNBb0033N16.14—Oryza sativa	71	0.021	9.08	Cell division cycle-associated protein—cell division
*RL versus RC 25*	Q6F367	Putative uncharacterised protein OJ1268_B08.14—Oryza sativa	60	0.27	11.94	Unknown—unknown
*RL versus RC 26*	P46801	60S ribosomal protein L16, mitochondrial—Oryza sativa	64	0.08	10.67	Translation, ribonucleoprotein, structural constituent of ribosome—protein synthesis
*RL versus RC 27*	Q93WC5	Pentatricopeptide repeat-containing protein At4g01990/T7B11_26, mitochondrial—Arabidopsis thaliana	60	0.22	6.59	Mitochondrion component, binding ability—mitochondrion component
*RL versus RC 28*	Q6UCC4	Maturase K (Fragment)—Planocarpa nitida	61	0.18	9.74	mRNA processing, chloroplast—mRNA processing
*RL versus RC 29*	Q8RVC8	Putative polyprotein (transposable element protein, putative)—Oryza sativa	61	0.22	8.69	Aspartic-type endopeptidase activity—peptidase
*RL versus RC 30*	Q3HVK7	Glycoprotein-like protein—Solanum tuberosum	82	0.017	10.44	Translation, ribosome, structural constituent of ribosome—protein synthesis
*RL versus RC 31*	Q2MGR2	SGS; HSP20-like chaperone—Medicago truncatula	56	0.55	6.98	Unknown—unknown
*RL versus RC 32*	Q9STH1	Stress-induced protein sti1-like protein—Arabidopsis thaliana	64	0.092	6	Response to hydrogen peroxide, heat and high light—response to oxidative stress,
*RL versus RC 33*	Q1SMU7	Hypothetical protein—Medicago truncatula	58	0.38	4.84	Unknown—unknown
*RL versus RC 34*	Q93WC5	Pentatricopeptide repeat-containing protein At4g01990/T7B11.26, mitochondrial—Arabidopsis thaliana	56	0.58	6.59	Mitochondrion component, binding ability—mitochondrion component
*RL versus RC 10a*	Q1SCC2	Cullin—Medicago truncatula	57	0.51	6.83	Unknown—unknown
*RL versus RC 10b*	Q3SC85	60S ribosomal protein L10—Lycopersicon esculentum	77	4.50E − 03	10.45	Translation, ribonucleoprotein, structural constituent of ribosome—protein synthesis
*RL versus RC 10c*	Q9MTH5	Putative membrane protein ycf1—Oenothera elata	55	0.78	9.92	Chloroplast membrane protein—photosynthesis
RL versus RC 18b	Q9MTH5	Putative membrane protein ycf1—Oenothera elata	68	0.041	9.92	Chloroplast membrane protein—photosynthesis
RS versus RC 168	B0BEM8	Cytochrome c biogenesis protein—Muraltia aspalatha	55	5.7	9.25	Chloroplast component, involves in respiratory chain complex IV assembly—respiratory
RS versus RC 214	Q6EPI0	Putative uncharacterised protein OSJNBb0057I13.23—Oryza sativa	57	2.2	4.47	Unknown—unknown
RS versus RC 242	A2T315	Photosystem I protein M—Angiopteris evecta	59	9.4E+02	5.8	Chloroplast component, plasma membrane-derived photosystem I component, involves in photosynthesis—photosynthesis
RS versus RC 296	C7A7K7	NBS-containing resistance-like protein—Corylus avellana	64	0.29	5.5	Have ATP-binding ability, involves in apoptosis and defence response (pathogen)—apoptosis and defence response to pathogen
	C5WNH9	Putative uncharacterised protein Sb01g038360—Sorghum bicolour	66	0.054	5.95	Have carboxy-lyase, magnesium ion binding and thiamin pyrophosphate binding activities—other metabolism (carboxy-lyase)
RS versus RC 298	Q49KX3	ATP-dependent Clp protease proteolytic subunit—Eucalyptus globulus	58	0.16	4.72	Cleaves peptides in various protein, play a role in degradation of misfolded proteins. Has a chymotrypsin-like activity—protease
**SL versus SC 43**	B6SSP2	EF hand family protein—Zea mays	52	4.9	5.63	Calcium ion binding—unknown
**SL versus SC 475**	B9T620	Putative uncharacterised protein—Ricinus communis	66	0.19	10.13	Unknown—unknown
**SL versus SC 116**	D7M917	Hypothetical protein ARALYDRAFT_914223—Arabidopsis lyrata	53	4.7	6.15	Protein phosphorylation—post-translational modification
**SL versus SC 707a**	C5X6P9	Hypothetical protein SORBIDRAFT_02g012940—Sorghum bicolour	70	0.087	6.54	GTPase activity—other metabolism (GTPase)
**SL versus SC 707b**	1GHSA(MSDB)	Glucan endo-1,3-beta-D-glucosidase (EC 3.2.1.39) II, chain A—Hordeum vulgare	53	12	8.83	Endoglucosidase—other metabolism (endoglucosidase)
**SL versus SC 75**	Q6Z0C7	Hypothetical protein OSJNBa0086M15.4 (Hypothetical protein OJ1465_C11.25)—Oryza sativa	56	0.58	11.2	Unknown—unknown
**SL versus SC 37**	C1FGT1	Predicted protein—Micromonas sp. (strain RCC299/NOUM17)	61	0.74	5.86	Nuclease activity, involves in nucleotide excision repair—DNA processing (nuclease/nucleotide excision repair)
**SL versus SC 69**	B9SAI3	Conserved hypothetical protein—Ricinus communis	59	10	5.8	Unknown—unknown
***SS versus SC 43***	Q8GUW5	Trehalose-6-phosphate synthase/phosphatase (Fragment)—Cypripedium parviflorum	65	0.078	7.84	Catalyse UDP-glucose and d-glucose 6-phosphate into UDP and alpha, alpha-trehalose 6-phosphate—regulates cell shape
***SS versus SC 179***	B8B946	Hypothetical protein OsI_30043—Oryza sativa	61	0.62	7.73	Unknown—unknown
***SS versus SC 692***	A9T965	Predicted protein—Physcomitrella patens	63	0.41	8.43	Have nucleic acid and zinc ion binding activities, bioprocess involved Unknown—unknown
***SS versus SC 159***	A2Q630	Phosphatidylinositol 3- and 4-kinase, catalytic—Medicago truncatula	56	1.9	5.76	Have kinase activity and phosphotransferase activity, alcohol group as acceptor—other metabolism (phosphotransferase)

Keys: (above proteins are arranged by treatments)

Italic: proteins up-regulated in Local tissues of Resistant line after 8-day aphid infestation

Normal: proteins up-regulated in Systemic tissues of Resistant line after 8-day aphid infestation

Bold: proteins up-regulated in Local tissues of Susceptible line after 8-day aphid infestation

Bold italic: proteins up-regulated in Systemic tissues of Susceptible line after 8-day aphid infestation

Please note: some proteins have more than one eligible ID, and it is not possible to distinguish which ID is true due to limitations of the approach

## Results

### Resistance of diploid wheat lines to grain aphid

A significant impact on the reproductive capacity of *S. avenae* was recorded when developing on different lines of *T. monoccoccum,* oats or hexaploid wheat (*F*_14,111_ = 4.67, *P* < 0.0001) (Fig. 1a). Whilst two diploid lines allowed aphids to produce in excess of 20 offspring per female, five lines only allowed for mean fecundities of less than 5 nymphs per female (Fig. 1a). By comparison, the hexaploid cultivar Claire was intermediate, with aphids producing an average of ca. 13 nymphs per female. Longevity closely reflected fecundity and was also significantly affected by variety (*F*_14,112_ = 3.78, *P* < 0.0001), such that aphids that produced the largest numbers of offspring typically exhibited the longest lifespans (Fig. 1b). Mean relative growth rate was similarly significantly affected by variety (Fig. 1c), although less markedly than fecundity or longevity (*F*_14,113_ = 8.32, *P* < 0.0001). Notably, the growth rate was highest on the commercial hexaploid wheat variety (Claire) and the habitual laboratory host, oats (Coast Black). These bioassays thus identified the following as exhibiting resistance to *S. avenae*: ACC16 INRA TM44, ACC17 PGR1752, ACC20 PGR1755, ACC26 PGR1761 and ACC27PGR1762, whilst ACC5 PGR1735 and ACC PGR1758 showed susceptibility to *S. avenae.* Based on these bioassays, the resistant diploid line (ACC20 PGR1755; #11) and the susceptible line ACC5 PGR1735 were selected in the present study to investigate genes and proteins potentially involved in the observed aphid resistance/tolerance.

### Proteome responses of wheat seedlings to aphid infestation

Local and systemic changes in the wheat leaf proteome in both a resistant diploid line (ACC20 PGR1755) and a susceptible diploid line (ACC5 PGR1735) in response to aphid infestation were investigated. Two time points were selected, 24 h (early response) and 8 days (late response). The proteome maps for 24 h and 8 days post-treatment control gels were consistent with previous 2-D gel separations reported for wheat and barley leaves (Geddes et al. 2008; Jiang et al. 2008; Ferry et al. 2011).

Proteome maps of wheat leaf tissues from each treatment (a resistant and a susceptible line) were compared with their respective controls using Progenesis SameSpots software (S Fig 1) and differentially regulated proteins identified by MS and MS/MS searches of nrNCBI/Swiss-Prot and a locally restricted wheat EST database. Differentially expressed proteins identified after 24 h and 8 days aphid infestation are presented in Tables 1 and 2, respectively. For both time points, both local and systemic responses were investigated. Approximately 200 protein spots were detected on each gel, with the differences in protein expression levels between a given line/time point/feeding site with its respective control varying from 4 to 34 protein spots. In the susceptible line, 16 protein spots were significantly up-regulated after 24-h aphid feeding, with 12 being involved in the local response and 4 involved in the systemic response, but after 8 days the up-regulated spots had decreased to 12 (8 locally and 4 systemically). In contrast, in the resistant line, 28 protein spots were significantly up-regulated after 24-h aphid feeding (17 locally and 11 systemically), and the number of up-regulated spots increased to 41 (37 locally and 4 systemically) after 8 days. Thus, the data show that more proteins were observed to be up-regulated in the resistant line at day 8 compared to the other treatments.

### Differentially expressed proteins in response to aphid infestation over time

**Fig. 2:**
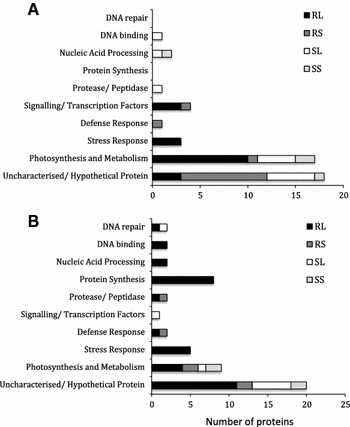
Changes in diploid wheat leaf proteins following aphid infestation for 24 h (a) or for 8 days (b). Proteins identified were assigned to categories based on biological process GO terms, shown as number of total response in each category for each treatment. Proteins that could not be identified were not included. First character: *R* resistant line, *S* susceptible line. Second character: *L* local tissues, *S* systemic tissues

**Fig. 3:**
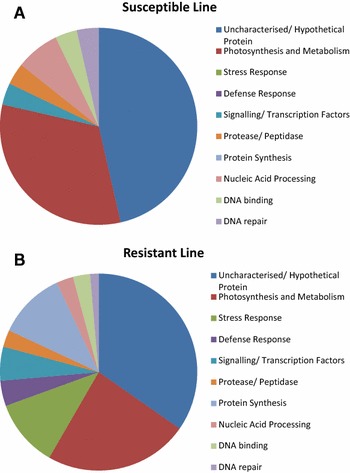
Global changes in wheat leaf proteins following aphid infestation of (a) the susceptible diploid line (ACC5 PGR#1735) and b the resistant diploid line (ACC20 PGR1755). Proteins identified were assigned to categories based on biological process GO terms, shown as number of total response in each category for *each line* at all time points tested. The results show that proteins involved in the stress response and the defence response were up-regulated in the resistant line, but only in response to aphid feeding. No such proteins were identified in the susceptible line either prior to, or following aphid infestation. (Color figure online)

Identified up-regulated protein spots listed in Tables 1 and 2 are grouped by functional categories in Fig. 2a, b. The results clearly show that the majority of identified proteins that were differentially regulated in both the resistant (ACC20 PGR1755) and susceptible (ACC5 PGR1735) diploid line following aphid infestation were involved in photosynthesis or other metabolic processes or were classified as being uncharacterised/unknown. Other proteins identified in both lines were shown to be involved in: ATP synthesis, proteolysis, post-translational modification, immune response, nuclear mRNA splicing, mRNA and miRNA processing, chromosome organisation, cell division and cytokinesis (Tables 1, 2). However, there were notable differences in the response of these two lines, particularly in respect of the up-regulation of general and specific stress response proteins. Importantly, these results show that proteins known to be involved in the stress response and the defence response were clearly induced in the resistant line, but not the susceptible line, in response to aphid infestation (Fig. 3a, b) and that different proteins were up-regulated at different time points. Thus, both spatial and temporal effects were observed. For example, at 24 h (representing an early response), a heat stress TF and dehydrin were up-regulated as part of the local response, whereas NBS–LRR type resistant protein was up-regulated as part of the systemic response. At 8 days (late response), a different set of proteins (two putative stress-induced protein sti1, putative viral resistance protein, MEDsa GLB1 and HSP20-like chaperone) was up-regulated as part of the local response, whilst a NBS-containing resistance-like protein was up-regulated as part of the systemic response. Additionally, at 24 h post-infestation, transcription factors and proteins with roles in signalling were shown to be clearly induced in the resistant line; by day 8, proteins involved in protein synthesis were shown to be up-regulated in this line. Interestingly, DNA repair proteins were also up-regulated in the resistant line. These results also show that the majority of the up-regulated proteins (in terms of number) were induced locally rather than systemically (Fig. 2a, b).

### General stress response proteins

The fold change (in normalised spot volume) for selected stress response proteins up-regulated in the resistant line in response to aphid infestation is presented in Table 3. The heat stress transcription factor A-5 (*Oryza sativa*) and dehydrin (*Nicotiana tabacum*) were up-regulated 2.6- and 2.9-fold, respectively, in the resistant line after 24-h aphid infestation (local response), as was a lipoxygenase (*Oryza sativa)*, a compound with multiple roles including plant response to stress, where the normalised spot volume showed a 3.2-fold increase. A putative stress-induced protein Sti1 (*Oryza sativa* and *Arabidopsis thaliana*), a non-symbiotic haemoglobin 1 MEDsa GLB1 (*Medicago sativa*) and a protein with similarity to guanylate binding proteins from *Arabidopsis* were also up-regulated in this line (3.5-, 3.7-, 2.8- and 2.2-fold increases, respectively), but only after 8 days aphid infestation (local response). In addition to these general stress response proteins, proteins with known roles in plant defence and apoptosis, such as NBS–LRR type resistance protein (*Oryza sativa*), were also shown to be systemically up-regulated in the resistant line after 24-h aphid infestation (2.4-fold increase). After 8 days of aphid feeding, a putative viral resistance protein (*Oryza sativa*) was identified as part of the local response in this resistant line (4.3-fold increase), whilst an NBS-containing resistance-like protein was identified as part of the systemic response (2.7-fold increase). Interestingly, proteins involved in protein synthesis and transcriptional regulation (ras-related protein Rab-18, *Zea mays,* 2.1-fold increase) were also up-regulated in the resistant line, but none were identified as being up-regulated in the susceptible line in response to aphid infestation (Fig. 2a, b; Table 3). Other proteins such as chaperones (SGS; HSP20-like chaperone, *Medicago truncatula,* 4.1-fold increase) and heat-shock proteins (sti 1) were also shown to be induced in the resistant line after 8 days post-aphid infestation. No stress/defence proteins were identified in the susceptible line in response to aphid infestation.

**Table 3:** Selected proteins (putative identity) known to be stress responsive

Predicted ID	Fold change
*Treatment*	
Resistant line (Local) 24 h	
Heat stress transcription factor A-5—Oryza sativa	2.6
Lipoxygenase—Oryza sativa	3.2
Dehydrin—Nicotiana tabacum	2.9
*Treatment*	
Resistant line (Systemic) 24 h	
NBS–LRR type resistance protein—Oryza sativa	2.4
Ras-related protein Rab-18—Zea Mays	2.1
*Treatment*	
Resistant line (Local) 8 days	
Similarity to guanylate binding protein—Arabidopsis thaliana	2.2
Putative stress-induced protein sti1—Oryza sativa	3.5
Putative viral resistance protein—Oryza sativa	4.3
Non-symbiotic haemoglobin 1, MEDsa GLB1—Medicago sativa	2.8
SGS; HSP20-like chaperone—Medicago truncatula	4.1
Stress-induced protein sti1-like protein—Arabidopsis thaliana	3.7
*Treatment*	
Resistant line (Systemic) 8 days	
NBS-containing resistance-like protein—Corylus avellana	2.7

Fold change relative to the appropriate control is shown

### Oxidative stress response proteins

Of particular interest, proteins associated with oxidative stress caused by insect herbivores were detected. After 24-h aphid infestation (local response), one stress response protein, called dehydrin from cultivated tobacco, and one redox protein from *Arabidopsis* (Table 1, spot RL v RC 1), were identified putatively in resistant plants, whilst after 8 days, a further three stress response proteins, putative stress-induced protein sti1 (from rice), non-symbiotic haemoglobin 1 (from alfalfa) and a stress-induced protein sti1-like protein (from *Arabidopsis*), which is involved in response to hydrogen peroxide and oxidative stress, were identified in these resistant plants. Surprisingly, no such protein was identified in the resistant plants as a systemic response, and neither were any such proteins identified in the susceptible plant line, irrespective of the time frame (24 h or 8 days).

### Proteins involved in photosynthesis and metabolism

Proteins involved in photosynthesis and metabolism formed the majority of proteins differentially expressed in either the susceptible or the resistant line in response to aphid infestation; interestingly, more proteins in these categories were detected after 24-h than 8-day aphid infestation (Tables 1, 2). Putative proteins involved in photosynthesis that were shown to be up-regulated locally in the resistant line at 24 h post-infestation included the large subunit of ribulose-1, 5-bisphosphate carboxylase/oxygenase and two ribulose bisphosphate oxygenase carboxylase activase from barley. Putative proteins involved in metabolism that were up-regulated locally at this same time point included a lipase class 3 family protein from rice (lipid metabolism), whilst a methyltransferase (Os06g0128100 protein from rice) was identified locally in these resistant plants, but after 8 days. Proteins with roles in protein degradation were also noted to be differentially regulated. For example, a protein with cysteine-type peptidase activity (OSJNBb0003B01.14 protein from rice) was identified putatively in the resistant line after 8 days aphid infestation as part of the local response, whilst a cell division protease (ftsH homolog 2 from rice), which is a probable ATP-dependent zinc metallopeptidase with hydrolase, metalloprotease and protease activities, was identified putatively as part of the local response in susceptible plants after 24-h aphid infestation.

### Proteins involved in transcriptional regulation

Proteins with roles in transcriptional regulation were also changed in response to aphid infestation, but predominantly in the susceptible wheat genotype. For example, a protein called At1g55310 from *Arabidopsis*, which is involved in nuclear mRNA splicing, was identified putatively in the susceptible line at 24 h post-local aphid infestation, whilst 5′-3′ exoribonuclease 2 (from *Arabidopsis)*, which is involved in mRNA processing and miRNA catabolic processes, was also identified putatively in these plants after 24 h, but only as part of the systemic response. Furthermore, a putative uncharacterised protein, OSJNBa0042F15.18 from rice, which is involved in DNA integration and RNA-dependent DNA replication, was identified putatively in the susceptible line after 24-h aphid local infestation. After 8d aphid infestation (local), two retrotransposon proteins, known to be involved in RNA-dependent DNA replication, were identified putatively in the resistant genotype after 24-h aphid infestation as part of the local response.

## Discussion

The preliminary bioassays carried out with diploid wheat lines and a standard commercial cultivar showed clear differences in susceptibility towards cereal aphids, although none of the lines tested showed strong resistance in the sense of causing significant levels of mortality of the pest. However, the partial resistance observed is sufficient to be useful in minimising cereal aphid outbreaks in the field by slowing the rate of population increase and thus provide a useful basis for further investigation. These results allowed two diploid wheat lines to be selected for a ‘resistant’ versus ‘susceptible’ comparison by proteomic analyses. The proteomics results showed that there were notable differences in terms of proteins up-regulated between the resistant and susceptible diploid wheat lines in response to aphid feeding. In this study, only up-regulated proteins were identified, as they are more likely to be directly involved in plant resistance to aphid infestation. Down-regulated proteins are likely to be the result of aphid manipulation or sabotage of plant defence (Smith and Boyko 2007).

Previous studies demonstrated that two-dimensional gel protein electrophoresis followed MALDI-TOF mass spectrometry (MS and MS/MS) was an effective initial tool for identifying differentially expressed proteins in wheat in response to aphid infestation (Ferry et al. 2011). Although the wheat genome has not been fully annotated, over 50 % of the protein spots excised from gels could be putatively identified as showing significant similarity to known wheat or other cereal/plant genes using NCBI, Swiss-Prot and wheat EST databases. Results from the previous study using the commercial line Claire (*T. aestivum*, hexaploid, A^u^A^u^BBDD) showed little resistance to aphid feeding, and thus, the diploid accessions were investigated as potential sources of aphid resistance genes/gene products (Diploid A^m^A^m^; *T. monococcum*) for exploitation in subsequent breeding programmes.

The present study could not definitively identify any specific gene-for-gene interactions between the diploid wheat *T. monococcum* and the grain aphid *S. avenae,* although proteins with similarity to NBS–LRR (nucleotide-binding site leucine-rich repeat) resistance proteins were identified as being systemically induced in the resistant line (ACC20 PGR1755) (Table 3). Most of the disease resistance genes (R genes) in plants cloned to date encode nucleotide-binding site leucine-rich repeat (NBS–LRR) proteins characterised by nucleotide-binding site (NBS) and leucine-rich repeat (LRR) domains. These abundant proteins are involved in the detection of pathogens (bacteria, viruses, fungi), nematodes and insects (McHale et al. 2006). Plant NBS–LRR proteins are numerous and are encoded by one of the largest gene families known in plants (Monosi et al. 2004). Disease resistance is the only function so far demonstrated for NBS–LRR proteins; however, a role in resistance has yet to be confirmed for most (Deslandes et al. 2002). Little is known about the regulation of the plant genes that encode NBS–LRRs. Consistent with the need for a rapid response to pathogen attack, many NBS–LRR-encoding genes are constitutively expressed at low levels in healthy, unchallenged tissue, although some show tissue-specific expression (McHale et al. 2006). They are upregulated, however, in response to bacterial flagellin, which induces basal resistance, suggesting that plants can establish a state of heightened sensitivity to pathogen attack (Zipfel et al. 2004). The tomato Mi-1 gene encoding an NBS–LRR-like protein confers resistance to both root-knot nematodes and potato aphids (Vos et al. 1998; Li et al. 2006), and aphid resistance in *Medicago truncatula* involves antixenosis and phloem-specific, inducible antibiosis, which maps to a single locus flanked by NBS–LRR resistance gene analogues (Klingler et al. 2005). Thus, findings from the present study provide the basis for a future molecular analysis of aphid resistance.

The diploid resistant line also responded to aphid infestation by significant up-regulation of stress response proteins, oxidative stress response proteins, defensive proteins and transcriptional regulators; interestingly, these same proteins were not detected in the susceptible line ACC5 PGR1735 in response to aphid infestation (Fig. 2a, b). This result suggests that the above proteins are playing a role in the observed resistance/tolerance of line ACC20 PGR1755 to aphid infestation. Furthermore, the diploid resistant wheat exhibited greater up-regulation of DNA synthesis/replication/repair proteins, which could have potential impact on plant resistance/tolerance to aphids and plant survival under aphid attack and oxidative stress.

Overall, the aphid responsive changes in the diploid wheat lines investigated were both spatially and temporally regulated, with differences between the two time points (24 h and 8 days), as well as differences in local and systemic responses (Tables 1, 2, 3; Fig. 2a, b). These proteins are grouped by functional categories (Fig. 3) to allow comparison between the resistant and susceptible lines and their potential roles discussed below.

### Metabolism related proteins

Plants experience a metabolic re-programming when attacked by herbivores; genes that are involved in photosynthesis may be up-regulated to compensate for nutritional loss caused by aphid attack (Smith and Boyko 2007). Photosynthetic adjustments in wheat can significantly contribute to its tolerance to damage/metabolic drain caused by Russian wheat aphid (RWA; *D. noxia*; Haile et al. 1999). In the present study, several enzymes involved in photosynthesis and ATP synthesis were up-regulated in response to aphid infestation (ribulose-1, 5-bisphosphate carboxylase/oxygenase large subunit, ribulose bisphosphate oxygenase carboxylase activase and ATPase alpha subunit) after 24-h infestation. Similar changes in metabolic processes were detected in both rice and barley following insect or pathogen attack, respectively (Wei et al. 2009; Geddes et al. 2008). Differential expression of photosynthetic or photorespiration genes have also been observed for *Myzus persicae* feeding on leaves of celery, *D. noxia* feeding on wheat foliage, *M. nicotianae* feeding on *Nicotiana attenuata* foliage, *Schizaphis graminum* feeding on *N. attenuata* foliage (Smith and Boyko 2007) and *Bemisia tabaci* feeding on *Arabidopsis* (Yin et al. 2012). These changes indicate the potential switch of plant metabolic resources from normal growth to defensive functions, when it is subjected to attack by phloem feeders. Thus, we also observe changes in protein turnover as plants adapt to different environmental conditions, including the challenge raised by insect pests (Pickart and Eddins 2004; Dreher and Callis 2007).

In the present study, a protein, OSJNBb0003B01.14 (Table 2, spot 20), with proteolysis/cysteine-type peptidase activity was found to be up-regulated locally after 8 days aphid infestation in the resistant line. Feng et al. (2003) showed similar results of regulated proteolysis of endogenous proteins can be a contributing factor to plant defence against insect pests. Similarly, changes in transcriptional regulators are essential to plants under different abiotic and biotic stresses. In this study, heat stress transcription factor A-5 (Table 1, spot RLvsRC2) and a Dof zinc finger protein (Fragment) (Table 1, spot RLvsRC8) were up-regulated locally in the resistant line 24-h post-aphid infestation. These findings are in agreement with those of other studies that suggest that transcription factors play an important role in plant response to environmental changes through regulation of plant signalling pathways (Lucyshyn and Wigge 2009; Koini et al. 2009; Casson et al. 2009). Metabolic reprogramming is further illustrated by results showing that aphid feeding up-regulates proteins involved in mRNA and miRNA processing (Fig. 2a, b), which in turn leads to changes in protein synthesis. Similar results have been shown for RWA feeding on wheat leading to the up-regulation of transcripts that are involved in protein synthesis (Boyko et al. 2006; Smith et al. 2010).

### Stress response and oxidative stress response proteins

Components of aphid salivary secretions are known to generate local and systemic production of reactive oxygen species (ROS; Tjallingii 2006), which is a commonly known early plant response to biotic stress (Apel and Hirt 2004; Bolwell and Wojtaszek 1997). ROS are important for plant signalling under insect attack, but can cause oxidative damage of membrane integrity due to lipid peroxidation, and can also generate highly cytotoxic compounds in the process. Therefore, plants have to enhance their resistance mechanisms, such as ROS scavenging and cell defence, to maintain homeostasis under stress conditions.

In this study, stress response proteins were identified as up-regulated in the resistant line after both 24-h and 8-day aphid infestation. A heat stress transcription factor A-5 (*Oryza sativa*, 24 h RL) and dehydrin (*Nicotiana tabacum*, 24 h RL) were induced early in the response, whilst after 8 days other known stress response proteins were induced including non-symbiotic haemoglobin 1, MEDsa GLB1 (*Medicago sativa*, 8 days RL) and two putative stress-induced protein sti1 (*O. sativa* and *Arabidopsis*, 8 days RL). The stress-induced protein sti1-like proteins (Table 3, induced in the resistant line as part of the local response, 8 days after infestation) are also thought to be induced in response to hydrogen peroxide and oxidative stress (Sano et al. 2013). Additionally, a hypothetical protein (*Arabidopsis*, 24 h RL, spot 1) is also thought to have a role in oxidative stress. These results indicate that diploid wheat plants recognised aphid infestation as a threat and a general stress response was triggered at or near the site of feeding, which was shown by the up-regulation of multiple stress-related proteins in leaves of the resistant line (24 h RL or 8 days RL). Interestingly, this stress response was not detected in the susceptible wheat line under the same treatment or the systemically infested resistant wheat leaves. This conclusion is further supported by the identification of induction of heat-shock proteins (HSPs) in the resistant line in response to aphid infestation. Stresses usually cause protein dysfunction, and maintaining functional proteins is particularly important for cell survival under stress. Many HSPs act as chaperones during protein folding, assembly, translocation and degradation, and can assist in protein refolding under stress conditions: for example, the HSP-70 chaperone system is crucial during both the stress response and development because protein misfolding and aggregation disrupt cellular homeostasis (Koizumi et al. 2014). They thus protect plants against stress by re-establishing normal protein conformation and hence cellular homeostasis (Wang et al. 2004). Similar results have been reported in other systems, for example, several proteins with putative functions in stress responses were identified in barley in response to *Rhopalosiphum padi* infestation (Gaupels et al. 2008). However, some chaperones are also multifunctional and may show enzymatic functions besides roles in protein folding (Scranton et al. 2012).

The induction of oxidative stress following aphid feeding is suggested by the identification of two stress-induced protein sti1-like proteins (Table 3) in this study and indeed in other aphid studies. Previous studies have shown that *D. noxia* infestation significantly induced an early accumulation of hydrogen peroxide and not only increased NADPH oxidase activity in a resistant (cv. Tugela DN) wheat line (Moloi and van der Westhuizen 2006), but also antioxidative enzyme activity in this same resistant wheat line compared to a susceptible wheat line (Moloi and van der Westhuizen 2008). Thus, the ability to control ROS levels in the wheat plant is closely linked to its resistance to aphid infestation. Oxidative stress is one of the first general reactions to the damage caused by phloem-feeding insects when they penetrate the plant (Wei et al. 2009). The expression of oxidative stress-related proteins, including a catalase, an ascorbate peroxidase and five extracellular class III peroxidases, was significantly increased in rice (*O. sativa*) in response to the brown plant hopper (*Nilaparvata lugens*; Wei et al. 2009). Proteomic studies carried out by Collins et al. (2010) revealed that proteins known to be involved in limiting ROS were more abundant in the resistant rather than susceptible varieties of *Arabidopsis* when under insect stress. Oxidative stress may also explain why proteins involved in DNA repair were identified in the present study. Interestingly, there are more DNA processing proteins up-regulated in the resistant line than the susceptible line following aphid feeding, suggesting some involvement of these proteins in the observed resistance/tolerance of line ACC20 PGR1755 to aphid infestation. Micro-array studies have similarly identified transcripts involved in DNA repair in *Arabidopsis* following aphid infestation, for example the increased expression of a nucleoside diphosphate-linked moiety transcript (Couldridge et al. 2007). Filkowski et al. (2004) found that mutant *Arabidopsis* lines impaired in certain aspects of protection against elevated levels of free radicals induce the production of scavenging enzymes earlier than wild-type plants. The higher levels of radical species resulted in the increased incidence of spontaneous double-strand breaks resulting in a higher expression of DNA repair genes. Gene sequences putatively involved in DNA synthesis were identified in wheat plants containing *Dn* genes in response to RWA biotype I attack (Boyko et al. 2006).

In addition to investigating both local and systemic responses in diploid wheat lines to aphid infestation, the present study also investigated this response over time to represent both an early response (24 h) and late response (8 days). Whilst stress and defence proteins were up-regulated at both time points, these represented different stress proteins, with more occurring during the late response phase. Interestingly, transcription factors and proteins with roles in signalling were shown to be induced during the early phase, as opposed to the late phase. Few studies have attempted to investigate these responses either over time or at the proteome level. Previous studies have shown that changes in transcript expression in wheat in response to RWA, where tolerance has previously been well characterised, comprise two phases: an immediate response (i.e. hypersensitive response) 24 h after infestation with RWA and a second prolonged response that prevails in the tissue for an extended period of time (i.e. systemic acquired resistance) (Botha et al. 2006), although differences in expression of transcripts were not reported. However, recently this same group has reported the differential regulation of transcripts involved in stress, signal transduction, photosynthesis, metabolism and gene regulation in susceptible wheat lines in response to RWA to better understand the different modes of resistance in wheat to this aphid species and the role of *Dn* genes (Botha et al. 2014). The ability of these genes to confer tolerance to *S. avenae* is currently being investigated.

Ultimately, the aim of the present study was to try to identify potential aphid resistance proteins. An NBS–LRR type resistance protein, types of which are known to be involved in apoptosis and plant defence (Takken and Joosten 2000; De Young and Innes 2006), was found to be up-regulated systemically in the resistant line following 24-h aphid infestation (Table 3). Furthermore, a putative viral resistance protein was shown to be up-regulated locally in the resistant line post 8 days aphid infestation as was the NBS-containing resistance-like protein (Table 3); however, in this case, the protein was systemically up-regulated. Both proteins have previously been shown to be involved in apoptosis and plant defence responses (Takken and Joosten 2000; De Young and Innes 2006; Rossignol et al. 2006). Possible involvement of these proteins in aphid resistance may occur, although further study is required to confirm and better understand their contributions to the observed resistance.

Currently, there is a major focus to identify potential aphid resistance genes through the study of aphid-induced gene expression (Thompson and Goggin 2006; De Vos et al. 2007; Smith et al. 2010). However, previous studies with commercial wheat lines have shown that this crop has a relatively poor inducible defence system (Ferry et al. 2011). Whilst there was very little evidence of any local induction of defensive proteins, e.g. proteinase inhibitors or other wound-responsive genes in response to grain aphid infestation (Thompson and Goggin 2006; Ferry et al. 2011) in this study, it does give new insights into the aphid-stress response in diploid wheat. The results suggest that stress response, oxidative stress response, defence response, apoptosis and DNA synthesis/replication/repair proteins play an important role in conferring resistance in the diploid line studied to *S. avenae*. This study indicates that the resistant diploid line ACC20 PGR1755 may provide a valuable resource in breeding wheat for resistance to aphids.

## Electronic supplementary material

Below is the link to the electronic supplementary material.

Supplementary material 1 Leaf proteome of resistant wheat (R, ACC20 PGR1755) following 24-h aphid infestation showing the local response versus non-infested leaf proteome of same aged resistant wheat plants. Up-regulated protein spots of twofold change or greater are indicated by O; down-regulated protein spots of twofold change or greater are indicated by O (PPT 3092 kb)
