# Supplementary material for: Proteomic analysis shows that stress response proteins are significantly up-regulated in resistant diploid wheat (Triticum monococcum) in response to attack by the grain aphid (Sitobion avenae)
Source: Mol Breed. 2015 Jan 28;35(2):57. doi: 10.1007/s11032-015-0220-x (PMC4308650; doi:10.1007/s11032-015-0220-x)
Supplement: Supplementary file 1 — Supplementary material 1 Leaf proteome of resistant wheat (R, ACC20 PGR1755) following 24-h aphid infestation showing the local response versus non-infested leaf proteome of same aged resistant wheat plants. Up-regulated protein spots of twofold change or greater are indicated by O; down-regulated protein spots of twofold change or greater are indicated by O (PPT 3092 kb) [file 11032_2015_220_MOESM1_ESM.ppt]

## Slide 1
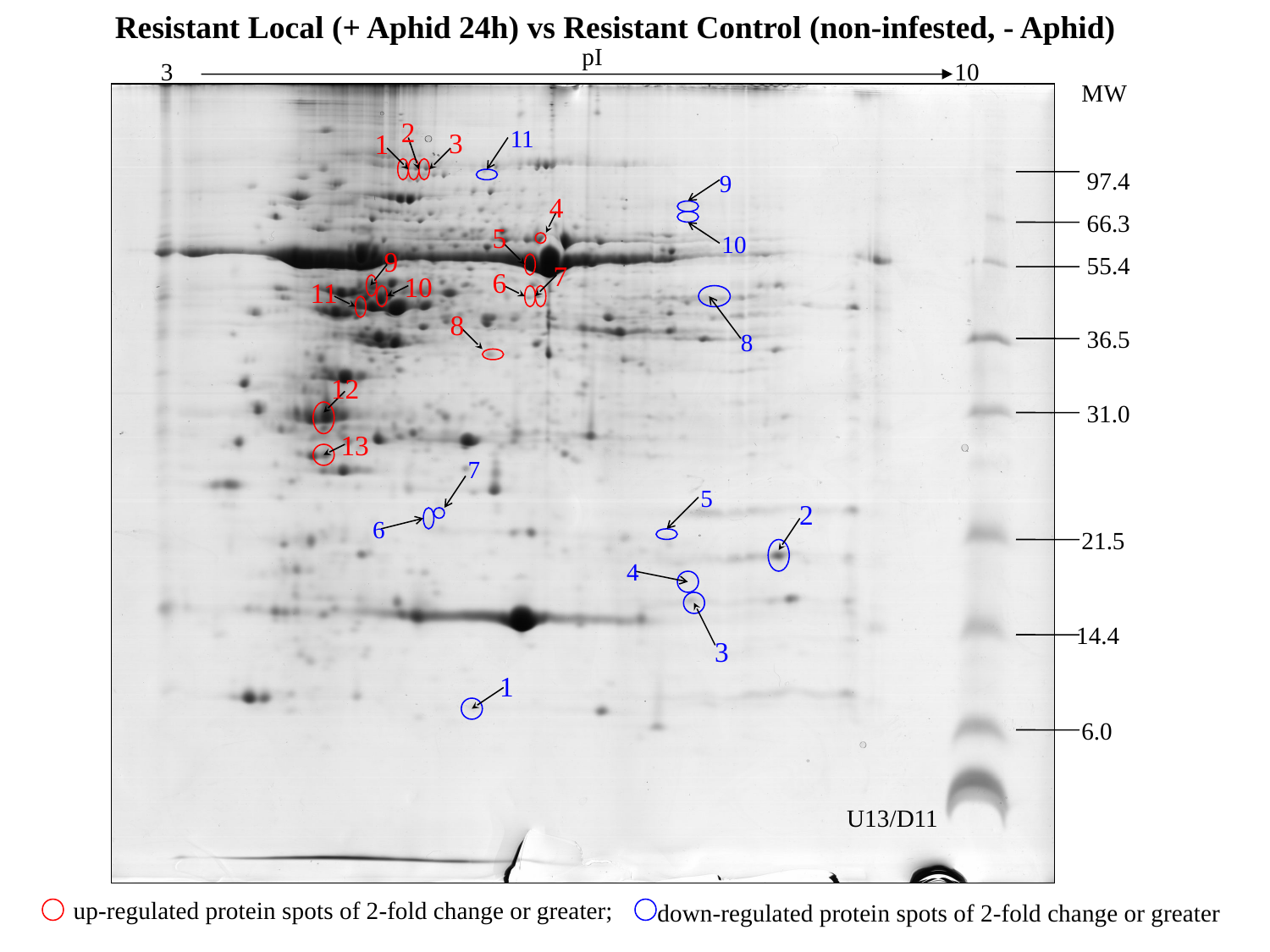

Resistant Local (+ Aphid 24h) vs Resistant Control (non-infested, - Aphid)
pI
3
10
MW
2
11
3
1
97.4
9
4
66.3
5
10
9
55.4
7
6
10
11
8
36.5
8
12
31.0
13
7
5
2
6
21.5
4
14.4
3
1
6.0
U13/D11
up-regulated protein spots of 2-fold change or greater;
down-regulated protein spots of 2-fold change or greater
